# Early Initiation of ARV During Pregnancy to Move towards Virtual Elimination of Mother-to-Child-Transmission of HIV-1 in Yunnan, China

**DOI:** 10.1371/journal.pone.0138104

**Published:** 2015-09-25

**Authors:** Kathrine Meyers, Haoyu Qian, Yingfeng Wu, Yunfei Lao, Qingling Chen, Xingqi Dong, Huiqin Li, Yiqing Yang, Chengqin Jiang, Zengquan Zhou

**Affiliations:** 1 Aaron Diamond AIDS Research Center, New York, New York, United States of America; 2 Yunnan AIDS Care Center, Kunming, Yunnan, China; 3 Linxiang Maternal and Child Hospital, Lincang, Yunnan, China; 4 Mangshi Maternal and Child Hospital, Dehong, Yunnan, China; 5 Yunnan AIDS Initiative, Kunming, Yunnan, China; University of North Carolina School of Medicine, UNITED STATES

## Abstract

**Objective:**

To identify factors associated with mother-to-child-transmission and late access to prevention of maternal to child transmission (PMTCT) services among HIV-infected women; and risk factors for infant mortality among HIV-exposed infants in order to assess the feasibility of virtual elimination of vertical transmission and pediatric HIV in this setting.

**Design:**

Observational study evaluating the impact of a provincial PMTCT program.

**Methods:**

The intervention was implemented in 26 counties of Yunnan Province, China at municipal and tertiary health care settings. Log linear regression models with generalized estimating equations were used to identify unadjusted and adjusted correlates for late ARV intervention and MTCT. Cox proportional hazard models with robust sandwich estimation were applied to examine correlates of infant mortality.

**Results:**

Mother-to-child- transmission rate of HIV was controlled to 2%, with late initiation of maternal ARV showing a strong association with vertical transmission and infant mortality. Risk factors for late initiation of maternal ARV were age, ethnicity, education, and having a husband not tested for HIV. Mortality rate among HIV-exposed infants was 2.9/100 person-years. In addition to late initiation of maternal ARV, ethnicity, low birth weight and preterm birth were associated with infant mortality.

**Conclusions:**

This PMTCT program in Yunnan achieved low rates of MTCT. However the infant mortality rate in this cohort of HIV-exposed children was almost three times the provincial rate. Virtual elimination of MTCT of HIV is an achievable goal in China, but more attention needs to be paid to HIV-free survival.

## Introduction

Mother-to-child-transmission of HIV (MTCT) during pregnancy, delivery, or breastfeeding is the major route of infection for the 330,000 children in resource-limited countries who continue to become infected each year. [1] Given the effectiveness of antiretroviral prophylaxis in reducing transmission of the virus from mother to child in 95–98% of cases [2–6] the global health discourse has progressed from the prevention of MTCT to its elimination (eMTCT). The World Health Organization’s (WHO) “Global Plan to eliminate HIV among Children under 15 and keeping their mothers alive” strives for a transmission rate below 5%, to ensure the children’s HIV-free survival, and to maintain their mother’s health. [7]

In China overall prevalence of HIV remains low, estimated at 0.06% in 2011. However, there is large regional disparity; 75% of the infections are reported within six provinces and Yunnan province alone comprises 21% of the national total.[8] The Bureau of Health estimates that there are approximately 1000 HIV-infected pregnant women delivering each year (unpublished data, Yunnan Bureau of Health) and has set ambitious targets to reach 95% with PMTCT services. In 2010 the National PMTCT Program released PMTCT guidelines calling for HAART for all pregnant women, in alignment with WHO’s Option B. Having piloted this approach in 13 counties through an international collaboration since 2006, the Yunnan Bureau of Health expanded the program to 26 counties in Yunnan that covered about half of reported HIV cases in the province. Building on the foundation of the national program, the international collaboration focused on capacity building of the local health system to facilitate rapid implementation and scale up of Option B. This paper reports on MTCT rates, HIV-exposed infant mortality, and the factors associated with these outcomes in the scale up of Option B in Yunnan Province between 2010–2012.

## Methods

### Site selection and study population

Twenty-six out of 119 counties in Yunnan Province were selected to participate in the program. County selection was based on HIV prevalence, existing infrastructure to provide treatment and PMTCT services, and local leadership commitment. All pregnant women visiting health facilities for ante-natal care (ANC) or labor and delivery (L&D) services were counseled and tested for HIV using the provider-initiated testing and counseling model. Blood samples from all women who screened positive for HIV were sent to the local CDC for Western Blot (WB) confirmation.

### Intervention

The PMTCT intervention for HIV-positive women and HIV-exposed infants followed the 2010 Chinese National PMTCT guideline. Briefly, HIV-positive pregnant women were advised to begin triple-ARV prophylaxis and counseled to remain on treatment after delivery for their own health benefit. HIV-exposed infants receive daily AZT or NVP from birth to 4–6 weeks age. No adjustments are made to infant prophylactic regimen based on maternal regimen. Exclusive formula feeding was recommended for HIV exposed infants, and the government provides a subsidy for infant formula.

### Testing algorithm

Blood samples are collected as early as six weeks and sent to the provincial laboratory for early infant diagnostics (EID). An HIV antibody test is administered at the 12-month visit, and repeated at 18 months if the 12-month test is reactive. Infants who are confirmed to be HIV-infected are referred to the local ARV clinic for treatment.

### Data collection

In Yunnan, all health facilities complete a standard registry for HIV counseling and testing of pregnant women and report the data to county MCH hospitals which in turn aggregate the data and report it to the provincial MCH. Case data for all HIV-positive women who gave birth between 2010 and 2012 in program counties was retrieved from the national PMTCT information management system, verified and cross-referenced with a physician’s report. Discrepancies were resolved through discussions with attending physician and local staff.

### Data analysis

The sample compositions of women and their HIV-exposed babies by demographic and intervention-related characteristics are reported. Late ARV intervention rate (%), MTCT rate (%) and infant mortality rate (per 100 person-years) was calculated for the whole sample and by demographic and intervention characteristics. Log linear regression models with generalized estimating equations (GEE) were used to identify unadjusted and adjusted correlates for late ARV intervention and MTCT. Sensitivity analysis with generalized linear mixed models (GLMMs) was conducted to confirm the parameter estimates. Cox proportional hazard models with robust sandwich estimation were applied to examine correlates of infant mortality. A *P* value less than 0.05 was considered statistically significant. Relative risk ratios and their 95% confidence intervals were estimated and reported for factors associated with late ARV intervention and MTCT. Similarly, hazard ratios and their 95% confidence intervals were estimated and reported for factors associated with mortality among HIV-exposed babies. Hospital cluster effects were adjusted for in all reported effect estimates. Kaplan Meier survival curves and log-rank tests were used to examine the effect of mothers’ ARV initiation on the mortality of HIV-exposed infants. We also calculated 9–24 months HIV-free survival rate among those who were followed up and tested for HIV during that period. The complete dataset and codebook are available in S1 File and S2 File. SAS 9.3 (SAS Institute, Research Triangle Park, NC, USA) was used to conduct the statistical analysis.

### Ethical approval

Ethical approval for the study was conferred by the Yunnan AIDS Care Center’s Institutional Review Board. All women consented to receive services provided by the program for themselves and on behalf of their children and approved the use of their de-identified data for research purposes.

## Results

A total of 1548 pregnant women across 26 counties of Yunnan Province were identified as HIV-infected between 2010 and 2012. Their demographic characteristics are presented in Table 1. Median age was 26 (IQR 23–30) and over half of the women identified as ethnic minorities (51.7%). The sample of women was overwhelmingly rural, with almost three-quarters of the women characterized as peasants (73.9%). Limited schooling was common with 12.1% reported having no education and a further 35.8% with no education beyond elementary school. The vast majority of women were married (95.7%). Among the 79.6% whose husbands had been tested, serodiscordance was high at 55.2%.

**Table 1:** Description of women is study sample (n = 1548).

Age (years)	n	%
Median (IQR)	26 (23, 30)	
15–20	145	9.4
21–25	524	33.9
26–30	501	32.4
31–35	254	16.4
>35	124	8.0
**Household registration**		
This county	1304	84.2
Other county in Yunnan	202	13.1
Other province	24	1.6
Other country	18	1.2
**Ethnicity**		
Han	748	48.3
Non-Han minority	800	51.7
**Occupation**		
Peasants	1142	73.9
Migrant workers	94	6.1
Housework or unemployed	216	14.0
Workers/white collar/other/unknown	96	6.0
**Marital status**	** **	** **
Married	1481	95.7
Living with partner	21	1.4
Single	39	2.5
Widowed/divorced	7	0.5
**Highest education level**		
No education	187	12.1
Primary school	553	35.8
Junior high	637	41.2
Senior high	131	8.5
College	38	2.5
**Year of enrollment**		
2010	505	32.6
2011	504	32.6
2012	539	34.8
**Husband/partner tested for HIV**		
Yes	1232	79.6
No	236	15.3
Unknown	80	5.2
**Husband/partner's HIV status (n = 1232)**		
Positive	530	43
Negative	680	55.2
Indeterminate	22	1.8
**Site of delivery**		
Municipal hospital	294	19.0
County hospital	1161	75.0
Township hospital	80	5.2
Home / other	13	0.8
**Mode of delivery**		
Emergency cesarean section	255	16.5
Elective cesarean section	428	27.7
Vaginal birth	865	55.9
**Number of births**		
1 baby	1534	99.1
Twins	14	0.9
**Maternal PMTCT regimen initiation**		
Pre-natal	1241	80.2
1st trimester	339	21.9
2nd trimester	639	41.3
3rd trimester	263	17.0
Labor and Delivery	246	15.9
No ART	61	3.9
**Maternal ARV regimen**		
cART during pregnancy	1041	67.3
scAZT	200	12.9
cART during L&D	229	14.8
sdNVP	17	1.1
No ARV during pregnancy or L&D	61	3.9

Notes: PMTCT prevention of mother-to-child transmission; L&D labor and delivery; cART combination antiretroviral therapy; ARV antiretrovirals; scAZT short course zidovudine; sdNVP single-dose nevirapine.

Ninety-four percent of women who were identified as HIV-infected during pregnancy delivered at county- or municipal-level hospitals, while the remainder delivered either at township clinics (5.2%) or at home (0.8%). Over 80% of women started ARV prophylaxis during pregnancy, and 67.3% started on combination ARV. A further 12.9% were given short-course AZT during pregnancy, 15.9% received ARVs during labor and delivery, and 3.9% did not receive any ARVs. (Table 1)

Infant outcomes are shown in Table 2. With 14 sets of twins, the total number of infants for whom outcomes were collected was 1562, including 9 (0.6%) stillbirths. Among the 1553 live births 6.4% were pre-term delivery and 12.6% were low or very low birth weight. Of these HIV-exposed infants 99.0% received ARV prophylaxis and 97.9% were exclusively formula-fed. Among infants who were tested for HIV (n = 1452, 93.6%), there was a two percent transmission rate (n = 29). Fifty-four (3.4%) HIV-exposed infants died, of whom 99% died within the first year of life, due to respiratory issues (27.8%), diarrhea (22.2%), or failure to thrive (16.7%). Out of 101 infants who were never tested for HIV, 42 (41.6%) died before they were tested, 30 (29.7%) were lost to follow up and 29 (28.7%) were followed but never tested. Using multiple imputation method to impute HIV status for the 101 (6.4%) who did not have HIV testing, the overall MTCT rises to 4% (95% CI 1.9–7.8), and for the non-ART intervention group, it is 10.4% (95% CI 4.2, 19.4).

**Table 2:** Infant outcomes (n = 1562).

	n	%
**Birth outcome**		
Still birth	9	0.6
Live birth	1553	99.4
pre-term delivery (< 37 weeks)	100	6.4
low birth weight (< 2.5 kg)	188	12.1
very low birth weight (<1.5 kg)	8	0.5
**Sex**		
Male	796	51.3
Female	757	48.7
**Infant prophylaxis**		
Yes	1537	99.0
No	16	1.0
**Ever tested for HIV infection**		
Yes	1452	93.6
No	101	6.4
Died too early for testing (≤ 3 months)	29	69.0
Died before testing (>3 months)	13	31.0
Lost to follow up	30	29.7
Followed but not tested	29	28.7
**Infected with HIV among tested**		
Yes	29	2.0
No	1423	98.0
**Infant mortality (excluding stillborn)**		
Total deaths	54	100.0
early neonatal (< 7 days)	14	25.9
late neonatal (7–28 days)	9	16.7
> 28 days but <1 year	29	53.7
≥ 1 year	2	3.7
**HIV status among deaths**		
HIV-positive	3	5.6
HIV-negative	9	16.7
Not tested	42	77.8
**Cause of death**		
Pneumonia / respiratory	15	27.8
Diarrhea	12	22.2
Low birth weight / early birth	9	16.7
Other/unknown	14	25.9

### Factors associated with MTCT

Table 3 shows crude and adjusted models exploring factors associated with MTCT. Results suggest starting ARV prophylaxis during the third semester (RR: 7.01 (3.31, 14.9)), during labor and delivery or not receiving ARV (RR: 7.55, (3.48, 16.4)) dramatically increase the risk for MTCT compared to those who initiated ARV in the first 28 weeks of pregnancy, regardless of maternal regimen. Babies who were not exclusively formula- fed were also at much higher risk of infection (RR: 4.12, (1.86, 9.10)).

**Table 3:** Factors associated with MTCT (n = 1452)^*^.

	Total n	%	HIV infected (n)	% MTCT	p-value	Unadjusted RR (95%CI)	Adjusted RR (95% CI)
**Age (years)**	** **	** **	** **	** **			
15–20	133	9.2	2	1.5		1	
21–25	495	34.1	10	2.0	0.80	1.36 (0.33, 5.52)	
26–30	477	32.9	12	2.5		1.73 (0.42, 7.09)	
31–35	232	16.0		1.7		1.19 (0.23, 6.02)	
>35	115	7.9	1	0.9		0.54 (0.12, 2.34)	
**Household registration**							
This county	1245	85.7	24	1.9		1	
Other county in Yunnan	177	12.2	4	5.4	0.83	1.13 (0.35, 3.67)	
Other province	20	1.4	1	5.0		2.42 (0.29, 20)	
Other country	10	0.7	0	0.0		0	
**Ethnicity**							
Han	707	48.7	12	1.7	0.43	1	
Non-Han minority	745	51.3	17	2.3		1.40 (0.69, 2.80)	
**Occupation**							
Peasants	1081	74.5	25	2.3		1	
Migrant workers	84	5.8	1	1.2	0.54	0.56 (0.09, 3.44)	
Housework or unemployed	197	13.6	2	1.0		0.43 (0.16, 1.18)	
Workers/white collar/other/unknown	90	6.2	1	1.1		0.50 (0.07, 3.24)	
**Highest education level**							
No education	167	11.5	3	1.8		1	
Primary school	514	35.4	16	3.1		1.61 (0.49, 5.32)	
Junior high	612	42.2	8	1.3	0.16	0.69 (0.21, 2.29)	
Senior high/college	158	10.9	2	1.3		0.64 (0.06, 6.55)	
**Husband/partner tested for HIV**							
Yes	1167	80.4	22	1.9	0.55	1	
No	217	14.9	5	2.3		1.19 (0.44, 3.19)	
Unknown	68	4.7	2	2.9		1.55 (0.36, 6.72)	
**Timing of PMTCT initiation**							
1st/2nd trimester	946	65.2	5	0.5	<0.001	1	1
3rd trimester	248	17.1	11	4.4		7.78 (3.65, 16.6)	**7.01 (3.31, 14.9)**
L&D or no ARV	258	17.8	13	5.0		9.3 (4.35, 19.9)	**7.55 (3.48, 16.4)**
**Site of delivery**							
Municipal hospital	261	18.0	5	1.9		1	1
County hospital	1106	76.2	18	1.6	0.007	0.88 (0.28, 2.76)	1.0 (0.34, 2.98)
Township hospital or home	85	5.9	6	7.1		3.8 (1.44,10.0)	**2.57 (1.03, 6.41)**
**Mode of delivery**							
Emergency cesarean section	231	15.9	4	1.7		1	
Elective cesarean section	423	29.1	6	1.4	0.5	0.86 (0.24, 3.05)	
Vaginal birth	798	55.0	19	2.4		1.43 (0.44, 4.61)	
**ARV regimen during pregnancy**							
cART	1002	69.0	13	1.3		1	
scAZT	192	13.2	3	1.6	<0.001	1.13 (0.27, 4.69)	
No ARV during pregnancy	258	17.8	13	5.0		3.85 (1.86, 7.98)	
**Baby sex**							
Male	753	51.9	11	1.5	0.13	1	1
Female	699	48.1	18	2.6		1.73 (0.94, 3.17)	1.56 (0.87, 2.80)
**Feeding mode**							
Formula feeding	1425	98.1	26	1.8		1	1
Mixed feeding	27	1.9	3	11.1	<0.001	5.91 (2.15,16.3)	**4.12 (1.86, 9.10)**
**Birth weight**							
<2.5kg	170	11.7	4	2.4		1.24 (0.49, 3.16)	
≥2.5kg	1281	88.3	25	2.0	0.74	1	
**Gestational age at birth**							
<37 weeks	87	6.0	3	3.5	0.32	1.83 (0.52, 6.44)	
≥37 weeks	1365	94.0	26	1.9		1	
**Total**	**1452**	**100.0**	**29**	**2.0**	(95% CI: 1.3, 2.7)		

*Analysis is limited to infants who have been HIV tested.

Notes: PMTCT prevention of mother-to-child transmission; L&D labor and delivery; CART combination antiretroviral therapy; ARV antiretrovirals; scAZT short course zidovudine; sdNVP single-dose nevirapine.

### Factors associated with infant mortality

**Fig 1:**
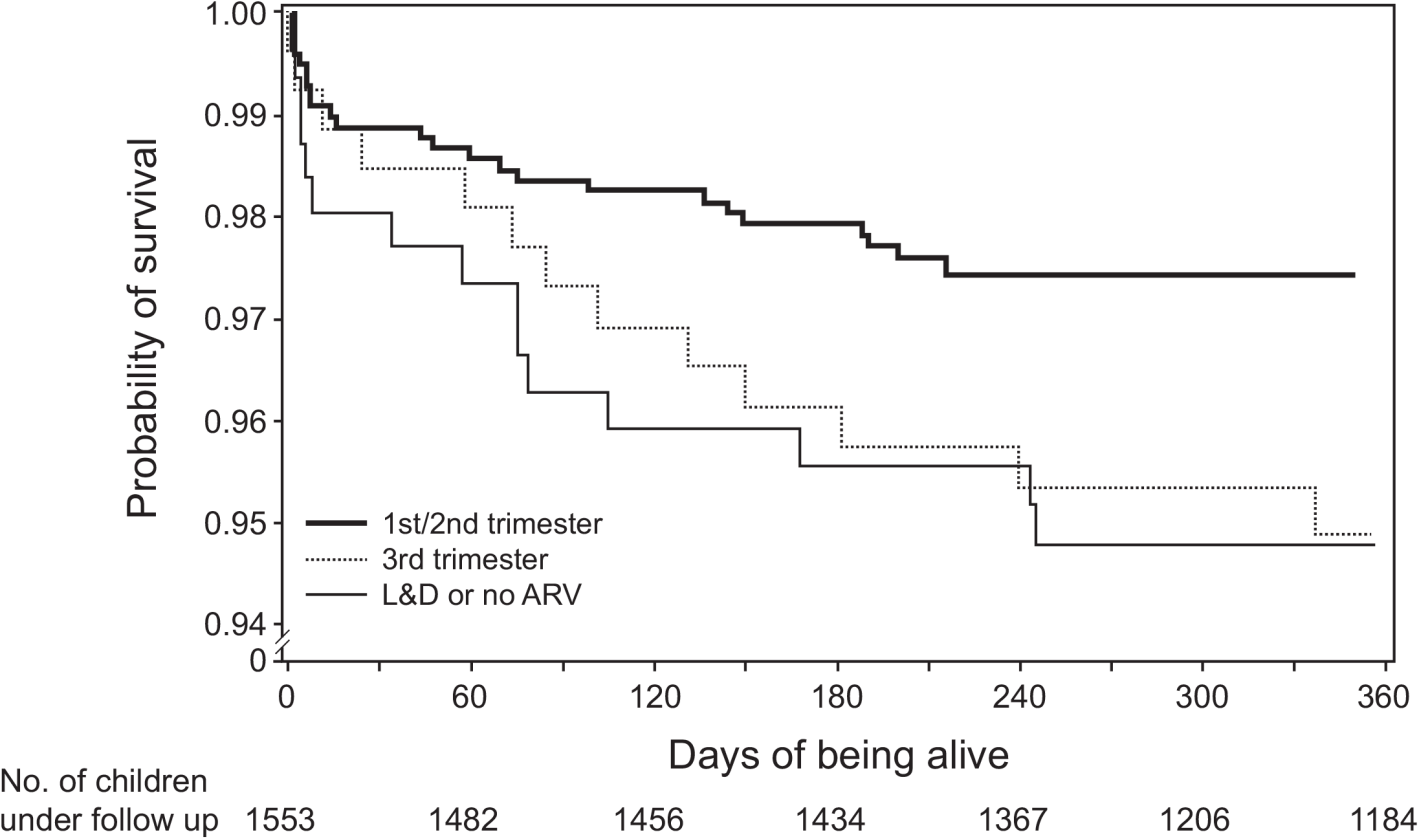
Kaplan-Meier survival curves by timing of maternal ARV initiation.

Mortality rate among HIV-exposed infants was 2. 9 /100 PY of follow up. Maternal factors associated with elevated mortality hazard ratios in the unadjusted model, shown in Table 4, included: ethnicity, timing of maternal ARV initiation and ARV regimen. Infant factors included birth weight and gestational age at birth, feeding mode, and whether the baby received prophylaxis. In the adjusted model infants born to ethnic minority women had significantly elevated hazard ratio compared to Han Chinese (HR = 2.67 (1.64, 4.38)), as did those with low birth weight (HR = 2.53 (1.41, 4.55)), and those born before 37 weeks (HR = 2.36, (1.1, 5.09)). A very high risk of death was found among infants who did not receive prophylaxis (HR = 7.7 (2.17, 27.4)), however, this should not be interpreted as causal. Rather, all but one of those who did not receive prophylaxis were pre-term births and died within hours of birth, making administration of the infant prophylaxis impossible. Mixed feeding was marginally associated with elevated risk of death (HR = 2.63 (1.16, 5.95) but was no longer significant after adjustment. Babies whose mothers initiated ARVs late in pregnancy also had higher mortality rates (4.4 / 100 PY compared to 2.0 / 100 PY among early interveners) and elevated hazard ratio of 2.23 (1.11, 4.38). Infant survival curves by timing of maternal ARV initiation are shown in Fig 1. The cumulative HIV-free survival rate of HIV-exposed children between 9–24 months of age is 94.3% (95% CI 93.1, 95.5).

**Table 4:** Factors associated with infant mortality (n = 1553).

	n	%	infant deaths (n)	Follow up (years)	Mortality rate (per 100 PY)	Unadjusted Hazard Ratio (95%CI)	Adjusted HR (95% CI)
**Maternal age**	** **	** **	** **	** **	** **		
15–20	144	9.3	7	172	4.1	1	
21–25	526	33.9	19	644	3.0	0.73 (0.39, 1.37)	
26–30	507	32.7	18	591	3.0	0.73 (0.32, 1.64)	
31–35	251	16.2	9	296	3.0	0.73 (0.36, 1.47)	
>35	125	8.1	1	148	0.7	0.16 (0.02, 1.24)	
**Household registration**							
This county	1311	84.4	46	1585	2.9	1	
Other county in Yunnan	200	12.9	6	228	2.6	0.86 (0.31, 2.39)	
Other province	24	1.6	1	27	3.7	1.23 (0.19, 7.97)	
Other country	18	1.2	1	9	11.1	2.27 (0.25, 21.1)	
**Ethnicity**							
Hanzu	749	48.2	13	908	1.4	1	1
Non-Han minority	804	51.8	41	945	4.3	2.99 (1.82, 4.91)	**2.68 (1.64, 4.38)**
**Occupation**	** **	** **	** **	** **	** **		
Peasants	1145	73.7	47	1387	3.4	1	
Migrant workers	96	6.2	0	104	0.0	0	
Housework or unemployed	217	14.0	5	250	2.0	0.58 (0.23, 1.45)	
Workers/white collar/other/unknown	95	6.1	2	109	1.8	0.51 (0.13, 1.99)	
Highest education level*							
No education	188	12.1	6	201	3.0	1	
Primary school	552	35.6	26	676	3.8	1.40 (0.71, 2.74)	
Junior high	643	41.5	20	765	2.6	0.97 (0.44, 2.15)	
Senior high/college	168	10.8	1	209	0.5	0.17 (0.02, 1.50)	
**Timing of PMTCT initiation**							
1st/2nd trimester	980	63.0	24	1179	2.0	1	1
3rd trimester	265	17.1	14	321	4.4	2.18 (1.12, 4.24)	**2.23 (1.11, 4.38)**
L&D or no ARV	308	19.8	16	349	4.6	2.24 (1.37, 3.69)	1.70 (0.87, 3.32)
**Site of delivery**							
Municipal hospital	294	18.9	12	333	3.6	1	
County hospital	1165	75.0	34	1405	2.4	0.70 (0.31, 1.61)	
Township hospital /home/other	94	6.0	8	113	7.1	2.08 (0.80, 5.39)	
**Mode of delivery**							
Emergency cesarean section	255	16.4	10	297	3.4	1	
Elective cesarean section	435	28.0	7	554	1.3	0.40 (0.15, 1.06)	
Vaginal birth	863	55.6	37	998	3.7	1.09 (0.64, 2.86)	
**ARV regimen during pregnancy**							
HAART	1045	67.3	33	1224	2.7	1	
scAZT	200	12.9	5	277	1.8	0.77 (0.28, 2.08)	
No ARV during pregnancy	308	19.8	16	349	4.6	1.73 (1.09, 2.73)	
**Baby sex**							
Male	796	51.3	24	968	2.5	1	
Female	757	48.7	30	882	3.4	1.33 (0.78, 2.28)	
**Feeding mode**							
Formula feeding	1518	97.8	51	1806	2.8	1	1
Mixed feeding	35	2.3	3	43	7.0	2.63 (1.16, 5.95)	1.47 (0.76, 2.84)
**Birth weight**							
≥2.5kg	1364	87.9	39	1646	2.4	1	1
<2.5kg	188	12.1	15	204	7.4	2.97 (1.99, 4.42)	**2.53 (1.41, 4.55)**
**Gestational age at birth**							
≥37 weeks	1453	93.6	44	1750	2.5	1	1
<37 weeks	100	6.4	10	100	10.0	3.66 (1.95, 6.89)	**2.36 (1.1, 5.09)**
**Infant prophylaxis**							
Yes	1537	99.0	50	1839	2.7	1	1
No	16	1.0	4	14	29.4	10.1 (3.58, 28.4)	**7.7 (2.17, 27.4)**
**HIV infection (among 1452 tested)**							
No	1423	98.0	9	1776	0.5	1	
Yes	29	2.0	3	33	9.2	3.16 (0.97, 10.2)	
Total	1553	100.0	54	1849	2.9	(95% CI: 2.2, 3.8)	

Notes: PMTCT prevention of mother-to-child transmission; L&D labor and delivery; CART combination antiretroviral therapy; ARV antiretrovirals; scAZT short course zidovudine; sdNVP single-dose nevirapine.

### Factors associated with late intervention

Given the association of early maternal ARV initiation with both preventing MTCT and infant mortality, we explored factors associated with late or no intervention, characterized as women who receive ARV prophylaxis after 28 weeks of pregnancy, including these who received ARV only during delivery and those who didn’t receive maternal prophylaxis. (Table 5) During the three-year program period, a total of 570 women (36.8%) received either late or no intervention.

**Table 5:** Factors associated with late initiation of maternal ARV.

	Total		Late Initiation of Maternal ARV		p-value	Unadjusted Relative Risk Ratio	Adjusted Relative Risk Ratio
	n	%	n	%			
**Age (years)**							
15–20	145	9.4	62	42.8		1	1
21–25	524	33.9	170	32.4	0.01	0.78 (0.63, 0.96)	**0.80 (0.73, 0.99)**
26–30	501	32.4	185	36.9		0.86 (0.72, 1.04)	0.86 (0.73, 1.03)
31–35	254	16.4	86	33.9		0.78 (0.60, 1.02)	0.80 (0.61, 1.03)
>35	124	8.0	67	54.0		1.23 (0.99, 1.52)	1.08 (0.84, 1.40)
**Household registration**							
This county	1304	84.2	467	35.8		1	
Other county in Yunnan	202	13.1	79	39.1	0.003	1.05 (0.86, 1.29)	1.07 (0.88, 1.30)
Other province	24	1.6	10	41.7		1.09 (0.58, 2.06)	0.99 (0.56, 1.72)
Other country	18	1.2	14	77.8		2.30 (1.92, 2.76)	**1.58 (1.17, 1.40)**
**Ethnicity**							
Han	748	48.3	238	31.8	<0.01	1	1
Non-Han minority	800	51.7	332	41.5		1.27 (1.11, 1.46)	**1.17 (1.04, 1.32)**
**Occupation**							
Peasants	1142	73.9	412	36.1		1	1
Migrant workers	94	6.1	42	44.7	0.05	1.24 (1.03, 1.48)	**1.33 (1.14, 1.55**)
Housework or unemployed	216	14.0	89	41.2		0.97 (0.71, 1.33)	1.06 (0.85, 1.31)
Workers/white collar/other/unknown	96	6.0	27	28.1		0.72 (0.45, 1.15)	0.89 (0.62, 1.26)
**Marital status**	** **	** **					
Married	1481	95.7	546	36.9		1	1
Living with partner	21	1.4	6	28.6	0.86	0.69 (0.42, 1.14)	
Single	39	2.5	15	38.5		1.03 (0.71, 1.51)	
Widowed/divorced	7	0.5	3	42.9		1.00 (0.39, 2.56)	
**Highest education level**							
No education	187	12.1	103	55.1	<0.01	1	1
Primary school	553	35.8	221	40		0.68 (0.59, 0.80)	**0.73 (0.64, 0.83)**
Junior high	637	41.2	197	30.9		0.53 (0.44, 0.63)	**0.58 (0.49, 0.69)**
Senior high/college	169	11.0	48	28.4		0.44 (0.31, 0.64)	**0.53 (0.39, 0.72)**
**Husband had been tested for HIV**							
Yes	1249	80.7	404	32.4	<0.01	1	1
No	236	15.3	136	57.6		1.68 (1.44, 1.96)	**1.57 (1.39, 1.78)**
Unknown	63	4.1	30	47.6		1.46 (1.14, 1.87)	**1.42 (1.13, 1.79)**
**Total**	1548	100	570	36.8			

Compared to those who were 20 or younger and older than 35, those between age 21 and 35 were less likely to have late ARV initiation (aRR between 0.79 to 0.84). Ethnicity appears to have an effect on timing of ARV initiation, with ethnic minorities having somewhat higher risk of initiating ARVs late in pregnancy (aRR = 1.18, 95% CI 1.03, 1.35). The effect of education was linear, with higher level of educational attainment associated with significantly lower odds of initiating ARVs late (aRR 0.49 for high school graduates, 95% CI 0.35, 0.67).

Migrant workers had higher risk of late ARV initiation in bivariate analysis (RR = 1.36, 95% CI 1.15, 1.62). Household registration appeared significant as well, particularly among foreign women residing in Yunnan, who had higher risk of late initiation (RR = 2.3, 1.92, 2.76). However these effects lost significance after adjustment.

Partner testing was also associated with timing of ARV initiation: 32.4% of women whose partner had been tested for HIV were late initiators as compared to 57.6% of those whose partner had not been tested (p = <0.01). Having a partner who did not get tested for HIV was associated with higher odds of late intervention for the woman (aRR = 1.59, 1.39, 1.81). Compared to 2010 and 2011, where 39.6% and 39.7% of women received intervention late, there seem to be an improvement in 2012, where the proportion of women who received late prophylaxis was reduced to 31.5%. (Data not shown).

## Discussion

This study has demonstrated that wide-scale implementation of triple-ARV in a rural, resource-limited region of China can be effective in controlling the vertical transmission rate to a level commensurate with elimination of pediatric HIV. Our findings are consistent with other studies that discussed the importance of early initiation of maternal ARV, both for prevention of transmission and for infant survival.[9–14]

### Individual-level factors

Individual-level factors (age, ethnicity, residency, and education) were strongly associated with the timing of maternal ARV initiation and additional research should be conducted to understand how exactly these factors affect initiation of ARV among mothers, in order to identify opportunities for intervention.

#### Ethnicity

The study points to the need for special efforts to communicate with ethnic minority women and effectively engage them in care. Our study enrolled women of close to a dozen ethnic minorities with variable levels of fluency in Mandarin and in the local provincial dialect, Yunnanese. A systematic review of utilization of maternal health care identified non-Han ethnicity as a recurrent factor associated with low utilization. [15] However, little is known, in our study or in the literature, about how a factor such as non-Han ethnicity operates as a barrier: is it because of communication difficulties between provider and client; because of discriminatory attitudes towards a minority group by an individual provider or a healthcare institution; or because of mistrust of the health system writ large by an ethnic minority woman. Only by understanding how each of these barriers operates in this context can programmatic adjustments be made in order to facilitate early intervention for all women.

#### Education

The linear association between higher educational attainment and higher odds of early ARV initiation is in line with studies that suggest a similar relationship between level of education and utilization of general and maternal healthcare of which ANC, HIV testing and ARV initiation are several examples. [15] Therefore, more attention needs to be paid to how to improve access to healthcare and increasing earlier intervention to HIV-infected women with less education.

#### Partner involvement and serodiscordance

While there is no provincial policy recommendation for partner testing in the context of prevention of MTCT programming, these results–a significantly higher proportion of women whose husbands have been tested for HIV initiating ARVs early–speak to the importance of partner involvement in PMTCT programming. [16] Others have shown the importance of male involvement in maternal regimen adherence, [17] supporting safe feeding practices, [18, 19] and decreased transmission and infant mortality.[19] The findings of this study are in line with this literature and suggest that partner testing should continue to be an important component of PMTCT programming. Research to understand more concretely how partner testing affects early ARV initiation among pregnant women could inform how such partner testing is conducted.

### Structural-level factors

#### Health care access

In China subsidized health care, including ANC, is most easily accessible in the township, county or municipality for which one has a residency permit (*hukou)*. In this study, women who accessed health care services in their place of residence accessed ARV earlier. Conversely, those who self-reported as migrant workers or as foreigners with no official residency permit in China, had higher probability of initiating ARVs later. The current health care financing system, in which access to health care and subsidies are tied to residency, creates a barrier to access for migrant workers and foreigners and may be the reason why a significantly higher proportion of non-local women accessed ARVs during labor and delivery than local residents (22.5% vs. 14.7%). Rural residents are insured through the New Rural Cooperative Medical Insurance, however migrant workers need to return to the place of their official residence to access this insurance.[20] Migrant women who are pregnant are likely to delay their return home in order to keep earning income for as long as possible, leading to late access to ante-natal care, including HIV testing. While the New Rural Cooperative Medical Insurance system has extended insurance coverage to millions of rural residents, the impact of this system on timely access to health care by migrant workers has been problematic. [20, 21]

### Infant outcomes

Infants received four to six week of AZT or NVP, regardless of maternal ARV regimen, as per Chinese national PMTCT guidelines. Given results from HPTN040 that demonstrated superior efficacy with a two or three-drug ART regimen for the prevention of intrapartum HIV transmission, [22] it may be advisable to consider adjusting the neonatal regimen for infants whose mothers did not receive ARVs during pregnancy.

Early initiation of ARVs was also associated with child survival. The literature has shown associations between improved maternal health and maternal survival and child survival. [11, 23] The lack of clinical markers documenting the mothers’ health is a significant limitation of this study (see below), but as other studies have shown, early initiation of ARVs results in suppressed viral loads by the time of delivery[23], which results in higher rates of infant survival. A secondary effect may be the quality of the provider-patient relationship. For example, a woman who has established and maintained a positive relationship with her health care provider throughout pregnancy may be more likely to seek care for a child who is failing to thrive either due to increased knowledge about signs of risk gained through her interaction with the health care provider or due to trust built up over time. A measure of the patient-provider relationship, such as the Patient-Provider Relationship Scale developed and tested within a PMTCT program in Pretoria, South Africa[24] would be a useful measure for future research to test this hypothesis.

While the program was able to limit the number of HIV-infections among infants, the mortality rate of HIV-exposed infants in our cohort is almost three times as high as infants of the general population in Yunnan. An analysis of the causes of death suggests that diarrhea and pneumonia, both commonly associated with formula feeding, led to a large proportion of deaths. So although formula-feeding was protective in terms of both HIV-infection and infant survival, systematic review of the case reports of infant deaths, which is currently done on an individual level, but not synthesized across cases, could yield important information about feeding practices, cleaning of feeding accouterments, and timeliness of health-care seeking for HIV-exposed babies and their relationship to mortality. While breastfeeding of HIV-exposed babies is not recommended in China, based on the stance that formula-feeding is acceptable, feasible, affordable, sustainable and safe almost everywhere in China, our data suggests that breastfeeding in the presence of continuing maternal ART (Option B+) could have a beneficial impact on infant mortality in this specific setting.

### Limitations

This is an observational study using case data collected through the maternal and child health system in Yunnan, and as such, cannot show causation. The lack of clinical data on the mothers, including CD4 counts, viral loads, and clinical staging, presents a limitation in the analysis. While CD4 and clinical staging are a routine component of HIV services in Yunnan, these data are reported in detail to the National AIDS Treatment Information Management System, but are incomplete in the National PMTCT Information Management System. Beyond being a limitation to the analysis in this research, the parallel reporting systems and the division of responsibilities for HIV-positive women’s care depending on whether they are pregnant or not is an issue impacting quality of care for HIV-positive women in Yunnan.

## Conclusion

This program was able to achieve a 2% transmission rate among the 95% of HIV-exposed babies who were tested. However, a further 3.4% of the cohort died. With performance targets for the national and provincial PMTCT program tied to limiting HIV transmission, not enough attention is paid to HIV-free survival. While this program demonstrates that virtual elimination of pediatric HIV is a plausible target in Yunnan, its achievement will only be meaningful if HIV-exposed infants’ mortality rates can be addressed at the same time, both at the level of the health care provider and at the policy level.

## Supporting Information

S1 File Complete Yunnan MTCT dataset. (SAV)

S2 File Yunnan MTCT codebook. (XLSX)
